# CNOT1 regulates circadian behaviour through *Per2* mRNA decay in a deadenylation-dependent manner

**DOI:** 10.1080/15476286.2022.2071026

**Published:** 2022-05-05

**Authors:** Haytham Mohamed Aly Mohamed, Akinori Takahashi, Saori Nishijima, Shungo Adachi, Iori Murai, Hitoshi Okamura, Tadashi Yamamoto

**Affiliations:** a Cell Signal Unit, Okinawa Institute of Science and Technology Graduate University, Okinawa, Japan; b Molecular Profiling Research Center for Drug Discovery, National Institute of Advanced Industrial Science and Technology, Tokyo, Japan; c Laboratory of Molecular Brain Science, Graduate School of Pharmaceutical Sciences, Kyoto University, Kyoto, Japan; d Department of Neuroscience, Graduate School of Medicine, Kyoto University, Kyoto, Japan

**Keywords:** Deadenylation, mRNA decay, circadian rhythm, post-transcriptional regulation

## Abstract

Circadian clocks are an endogenous internal timekeeping mechanism that drives the rhythmic expression of genes, controlling the 24 h oscillatory pattern in behaviour and physiology. It has been recently shown that post-transcriptional mechanisms are essential for controlling rhythmic gene expression. Controlling the stability of mRNA through poly(A) tail length modulation is one such mechanism. In this study, we show that *Cnot1*, encoding the scaffold protein of the CCR4-NOT deadenylase complex, is highly expressed in the suprachiasmatic nucleus, the master timekeeper. CNOT1 deficiency in mice results in circadian period lengthening and alterations in the mRNA and protein expression patterns of various clock genes, mainly *Per2. Per2* mRNA exhibited a longer poly(A) tail and increased mRNA stability in *Cnot1^+/−^* mice. CNOT1 is recruited to *Per2* mRNA through BRF1 (ZFP36L1), which itself oscillates in antiphase with *Per2* mRNA. Upon *Brf1* knockdown, *Per2* mRNA is stabilized leading to increased PER2 expression levels. This suggests that CNOT1 plays a role in tuning and regulating the mammalian circadian clock.

## Introduction

Behavioural, physiological, and cognitive cycles such as sleep–wake, feeding–fasting, activity–rest, hormone secretion, and energy metabolism are controlled by an endogenous internal timekeeping mechanism referred to as the circadian clock. This vital biological timing system is conserved throughout the phylogenetic tree from unicellular organisms such as cyanobacteria to plants and mammals. It provides organisms with the ability to quickly anticipate, adapt, and coordinate their biology at a molecular level by regulating gene expression, and consequently their behaviour to that of the constantly changing environment [1,2]. The master circadian clock in the hypothalamic suprachiasmatic nucleus (SCN) synchronizes to external environmental cues, *Zeitgeber*, primarily light, that arise from the predictable daily changes in the light–dark cycles [3]. This results in rhythmic processes on a biochemical, molecular, and behavioural level with a periodicity of 24 hours [4,5].

The basic molecular clock mechanism in the majority of cells consists of a network of transcriptional-translational autoregulatory loops (TTLs), which drive the rhythmic expression of the core clock components [6]. The core TTL of the molecular clock consists of four integral proteins: two activators (CLOCK and BMAL1) and two repressors (PER and CRY). CLOCK and BMAL1 are basic helix-loop-helix (HLH), period-Arnt-single-minded (PAS) transcription factors that heterodimerize and bind to the E-box *cis* regulatory enhancer sequences within the promoters of the repressor genes *Per* (*Per1, Per2, Per3*) and *Cry* (*Cry1* and *Cry2*), as well as other clock-controlled output genes (CCGs), activating their transcription. Upon transcription, *Per* and *Cry* mRNAs are translated in the cytoplasm and form multimeric protein complexes [7]. As soon as sufficient amounts of PER and CRY proteins have accumulated, they are phosphorylated by the casein kinases CK1δ/ε and translocate into the nucleus [8,9]. Once in the nucleus, the PER/CRY heterodimer represses the activity of the BMAL1/CLOCK complex and inhibits further transcription of their own genes as well as other CCGs [10–13]. The repression on BMAL1/CLOCK complex is relieved as PER and CRY proteins are degraded by ubiquitin-dependent pathways. A second TTL mechanism exists through another family of CCGs that are controlled by the BMAL1/CLOCK complex – nuclear receptors REV-ERB (REV-ERBα and REV-ERBβ), and retinoic-acid-related orphan nuclear receptors (RORs). RORs and REV-ERBs bind to the promoter of *Bmal1*, with ROR binding activating transcription of *Bmal1*, whereas REV-ERB inhibits transcription. Together, these two TTL loops operate to regulate the transcription of core clock genes, with a period of approximately 24 hours, and form the molecular basis for the autonomic clock found in mammalian cells [7].

Transcriptomic studies have suggested that over 50% of mouse genes are rhythmically expressed in at least one tissue, and up to 20% of mRNAs are expressed rhythmically in the mouse liver [14–17]. These oscillations were thought to be driven solely by the rhythmic changes in transcription activation/repression cycles controlled by transcription regulators. However, it has been shown that rhythmic *de novo* transcription accounts for only 22% of rhythmic mRNAs, with the remaining 78% of rhythmic mRNAs controlled via an unknown mechanism [18–20]. Furthermore, up to 50% of rhythmic proteins exhibit non-rhythmic mRNA expression [21,22]. This inconsistency between mRNA and protein expression is not a new idea, given that the correlation between them in general can be as low as 40% [23]. Therefore, an important regulatory step between the transcription of mRNAs and protein expression might explain the relationship between mRNA levels and rhythmic protein expression or lack thereof. One such mechanism is mRNA stability/decay control. It has been estimated that up to 30% of rhythmic mRNA in the liver is a result of mRNA degradation [24,25]. In eukaryotes, there are two main conventional mRNA decay pathways that are both dependent on deadenylation, that is, the shortening of the poly(A) tail [26]. The process of deadenylation of an mRNA results in translation suppression, destabilization, and subsequent degradation [27,28]. The rate-limiting step of mRNA decay is deadenylation [27]. In mammals, this process is mediated by 10 known deadenylases, four of which are incorporated into a major cytoplasmic deadenylase complex, the CCR4-NOT complex [29]. The CCR4-NOT complex has been implicated in various cellular processes including cell growth, DNA repair, mRNA export, as well as in a physiological role in regulation of bone mass, spermatogenesis, energy expenditure, and heart function [30–33]. CNOT1 acts as a scaffold protein, maintaining the integrity and function of the complex as a whole [34]. Downregulation of CNOT1 leads to elongation of mRNA poly(A) tails and disrupts deadenylase activity of the complex [34,35]. Therefore, we hypothesized that, since the CCR4-NOT complex is a major regulator of mRNA metabolism, it should be involved in regulating circadian rhythms.

In this study, we examined the role of the CCR4-NOT complex in regulating circadian behaviour by focusing on the functional role of CNOT1. We found that *Cnot1* mRNA is highly expressed in the mouse SCN and that CNOT1 deficiency in mice resulted in a lengthening of the circadian period owing to a delay in *Per2* decay and consequently a delay in PER2 levels. We show that CNOT1 binds to *Per2* via the RNA-binding protein (RBP) BRF1 and destabilizes it through shortening of its poly(A) tail. Our study suggests that CNOT1 plays a role in tuning and regulating the mammalian circadian clock and circadian behaviour.

## Results

### *Cnot1* is under circadian control in the liver

**Figure 1.**
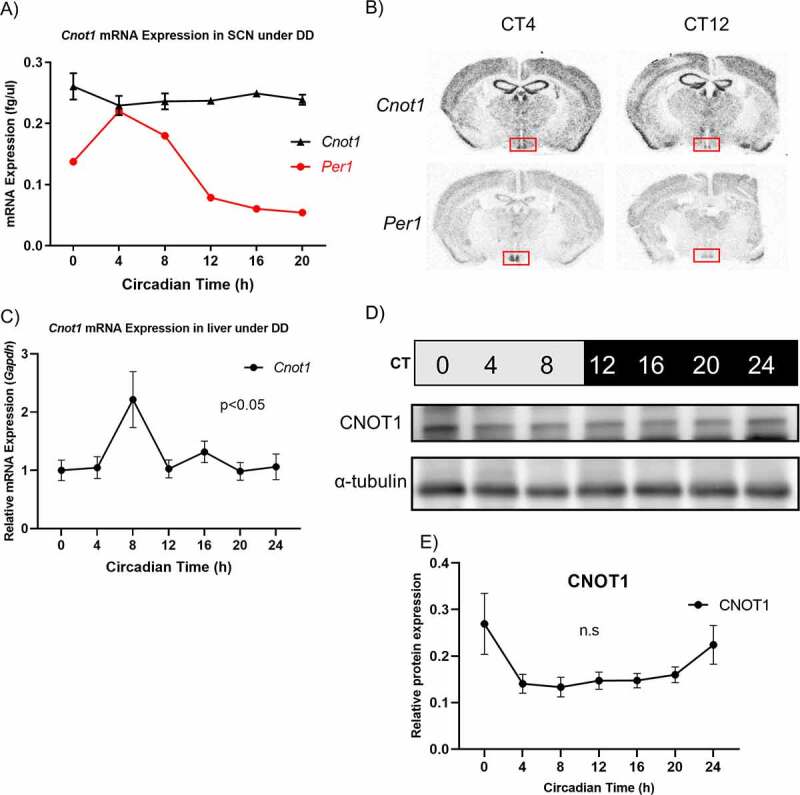
CNOT1 gene expression under constant darkness (DD).

To investigate the role of mRNA decay in regulating the circadian rhythm, we focused on CNOT1, the scaffold protein of the CCR4-NOT deadenylase complex, which is essential for maintaining the structural integrity and functional activity of the complex [33,34]. We previously showed that CNOT1 is expressed in the liver and brain, but whether the expression changes in a time-dependent manner is not known [36]. To determine the circadian expression of *Cnot1* in the SCN and liver, mice were sacrificed at 4-hour intervals around the clock under constant darkness (DD) and RNA was extracted from the SCN and liver and analysed by quantitative PCR (qPCR). In the SCN *Cnot1* mRNA levels were constant throughout the circadian day, compared to the oscillating *Per1* (Fig. 1A). To further confirm the observed expression pattern, we examined the expression of *Cnot1* and *Per1* using [33P] radiolabeled UTP *in situ* hybridization at two different circadian time (CT) points: CT4 and CT12, where CT0 indicates the beginning of the subject day (7am) and CT12 is the beginning of the subjective night (7pm). *Cnot1* was expressed at both time points in the SCN with no time-dependent difference as well as other brain areas including the cerebral cortex, the piriform cortex, cortical amygdala, and the hippocampus (Fig. 1B). *Cnot1* expression in the SCN suggests that it may be involved in regulating circadian behaviour.

A) Absolute quantification measured by qPCR of *Cnot1* mRNA expression in the SCN under DD conditions normalized against *36B4. Per1* is a cloned plasmid DNA used as standard for comparison. Values are means ± SEM; *n* = 4–5. B) [33P] UTP radiolabeled *in situ* hybridization showing *Cnot1* and *Per1* expression in WT mouse SCN collected under constant darkness at CT4 and CT12. The red box indicates the location of the SCN. C). Relative mRNA expression of *Cnot1* in the liver under DD conditions normalized against *Gapdh* and *36B4* using the ΔΔCT method. Values are means ± SEM; *n* = 3–5. D) Total protein lysates immunoblotted for CNOT1 and α-tubulin (loading control) in the livers of mice under DD conditions. E) Relative protein quantification of CNOT1. One-way ANOVA was used to assess rhythmicity. (N.S: not significant).

In contrast to the SCN, *Cnot1* mRNA exhibited a clear circadian rhythm in the liver (p < 0.05) with peak expression at CT4-8 and a nadir at CT20-24, with an approximately 2-fold difference in amplitude (Fig. 1C), while CNOT1 protein levels remained constant (Fig. 1D-E). Taken together, these data suggested that in the liver but not in the SCN, *Cnot1* expression is under circadian control.

### CNOT1 deficiency elongates circadian period

Based on the initial observation that *Cnot1* is expressed in the SCN, we examined whether CNOT1 regulates the circadian clock *in vivo* by employing the use of *Cnot1* knockout (KO) mice. Whole-body *Cnot1* KO mice are embryonically lethal at E6.5 and therefore we used heterozygous *Cnot1-*deficient (*Cnot1*^+/−^) mice [37]. This is the first study in which *Cnot1*^+/−^ mice are used to analyse the function of CNOT1 in physiological conditions. Upon handling the mice, we noticed that there was a significant difference in body size between *Cnot1*^+/−^ mice and their wild-type (WT) littermates; *Cnot1*^+/−^ mice exhibited a decrease in total body weight (Supplementary Figure 1A–B). To confirm that CNOT1 protein levels were reduced in *Cnot1*^+/−^ mice, we performed immunoblotting against CNOT1 using *Cnot1*^+/−^ and WT liver lysate, which showed that the CNOT1 level was reduced to approximately 45% (Supplementary Figure 1C–D). Furthermore, we assessed whether other components of the CCR4-NOT complex were affected by the reduction of CNOT1 and observed that only CNOT8 and CNOT9 were reduced (Supplementary Figure 1E-F).

**Figure 2.**
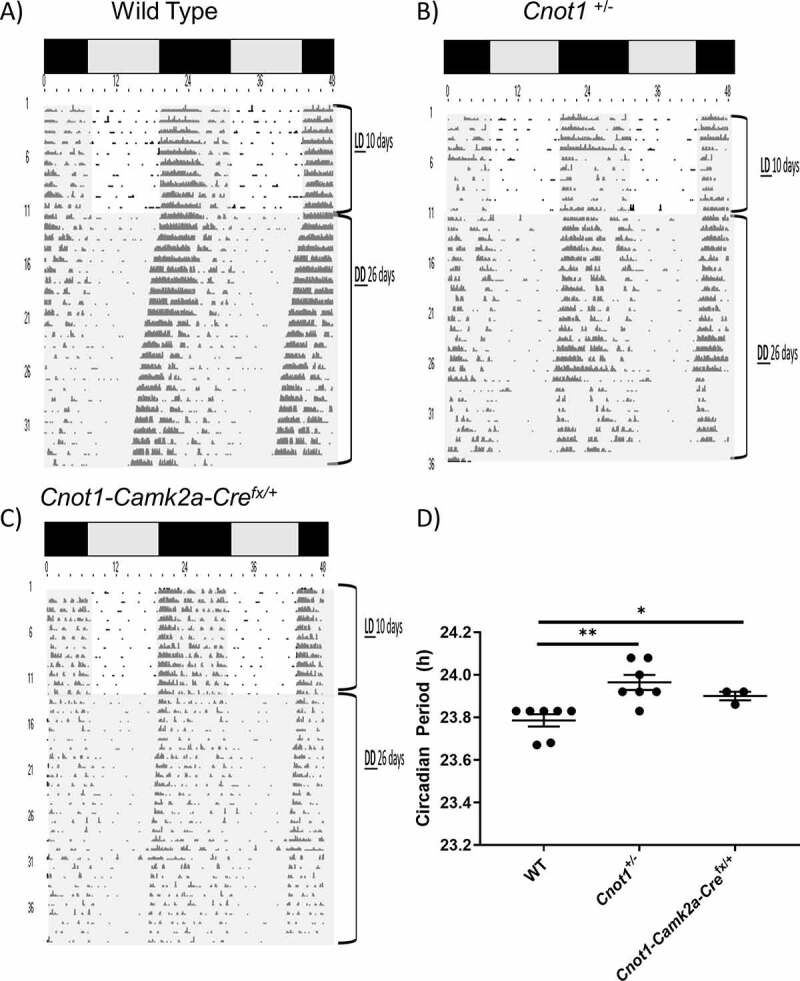
CNOT1 deficiency elongates circadian period.

To determine the effects of CNOT1 reduction on behavioural rhythmicity, we measured the wheel-running activity of *Cnot1*^+/−^ mice and their WT littermates. Mice aged 6–8 weeks were initially housed in cages equipped to measure wheel-running activity for 10 days in a 12-h light:12-h dark (LD) cycle and then transferred under DD for at least 21 days, where the endogenous circadian period can be revealed. WT and *Cnot1^+/-^* mice showed robust wheel-running activity rhythms under a LD cycle and exhibited normal entrainment to light as evident by activity onset at the beginning of the night (Fig. 2A,B). When transferred to DD condition, WT mice activity onset occurred earlier each successive day, while *Cnot1^+/-^* activity onset did not change. In DD conditions, *Cnot1*^+/−^ mice displayed a longer circadian period (23.96 ± 0.04 h, n = 7) than their WT littermates (23.78 ± 0.03 h, n = 7; p < 0.01) (Fig. 2D).

Representative double-plotted actograms of A) Wild-type; B) *Cnot1*^+/−^, and C) *Cnot1-CamK2a-Cre^f^*^x/+^ mice housed in wheel running cages for at least 30 days. The 10 days LD period and the 26 days DD period is labelled to the right of the actogram. Under both LD and DD conditions, time of day is represented by grey and black panels above the actogram as well as in white and grey shading that indicates periods of day and night, respectively. D) Plotted are the period lengths of individual animals (means ± SEM) of WT, *Cnot1*^+/−^, and *Cnot1-CamKII-Cre^f^*^x/+^ mice (n = 7 for WT and n = 7 for *Cnot1*^+/−^, and n = 3 for *Cnot1-CamKII-Cre^f^*^x/+^, **p < 0.01, unpaired t-test).

To rule out that the observed change in circadian behavioural rhythm was not due to side-effects of whole-body heterozygosity, we generated *Cnot1-Camk2a-Cre^f^*^x/+^ mice. *Camk2a* is expressed mainly in neurons in the forebrain, including the SCN [38]. *Camk2a-Cre* mice were crossed with *Cnot1* conditional KO mice (*Cnot1^fx^*^/fx^) mice to generate *Cnot1-Camk2a-Cre^f^*^x/+^ mice. *Cnot1-Camk2a-Cre^f^*^x/fx^ mice were not used because they exhibited early postnatal lethality primarily owing to improper formation of the forebrain (unpublished data). *Cnot1-Camk2a-Cre^f^*^x/+^ mice were tested for circadian deficiency, and they exhibited a longer circadian period (23.9 ± 0.02 h, n = 3; p < 0.05; Fig. 2C, D) as observed in *Cnot1^+/-^* mice. This clearly indicated that CNOT1 deficiency affects circadian behaviour, as exhibited by a longer circadian period length.

### Molecular clock gene expression is altered in *Cnot1*^*+/−*^ mice

**Figure 3.**
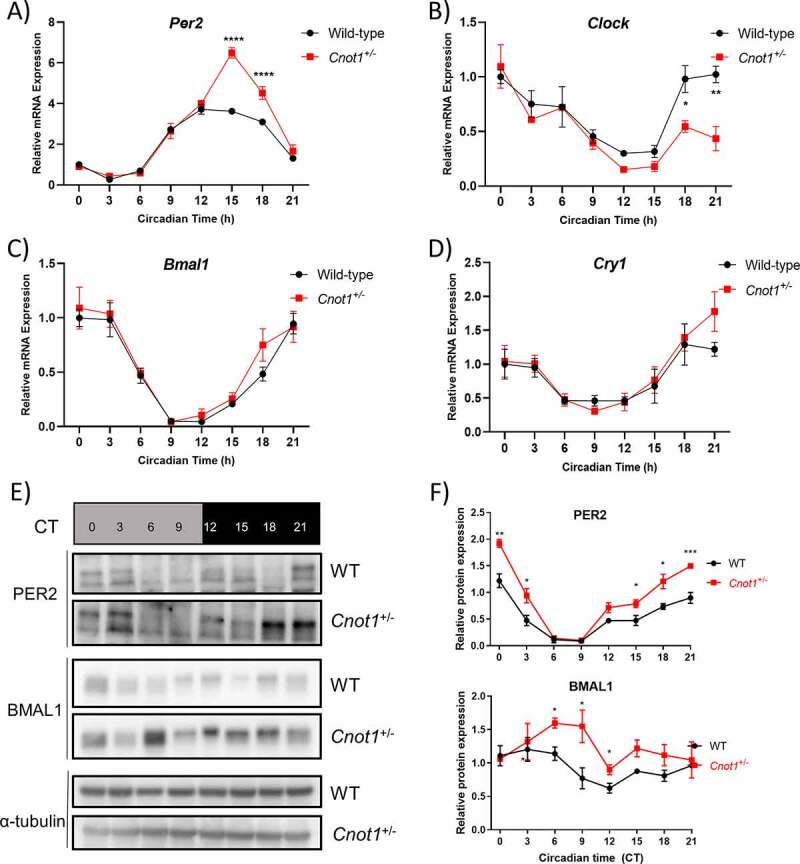
Molecular clock gene expression is altered in *Cnot1*^*+/−*^ mice.

To delineate the molecular mechanism by which a reduction in CNOT1 leads to an elongated circadian period, we examined the circadian mRNA profile of canonical clock genes ‒ *Per2, Bmal1, Cry1*, and *Clock* ‒ in liver tissue collected every 3 h under DD using qPCR (Fig. 3). In *Cnot1^+/−^* livers, *Per2* mRNA levels retained their circadian rhythmicity but exhibited a ~ 3 h phase delay in peak expression at CT15 compared with that in livers from WT mice at CT12 (Fig. 3A). Moreover, *Per2* mRNA expression was elevated in *Cnot1^+/−^* mice during the subjective night (CT15-18; Fig. 3A). *Clock, Bmal1*, and *Cry1* mRNAs all retained their circadian rhythmicity with minor changes in expression amplitude, except for *Clock*, which showed a significant reduction in levels during the subjective night (Fig. 3B–D).

Relative mRNA circadian expression of A) *Per2*; B) *Clock*; C) *Bmal1*, and D) *Cry1* in WT (black curve) and *Cnot1^+/−^* (red curve) in mouse liver normalized against *Gapdh* using ΔΔCt method. E) Total protein lysates from mouse liver under DD were immunoblotted against PER2, BMAL1, and α-tubulin. F). Relative protein expression of PER2 and BMAL1 normalized against α-tubulin. Values are mean ± SEM, n = 3–5. Two-way ANOVA was used followed by Sidak’s multiple comparison test. *p < 0.05; **p < 0.01, and ***p < 0.001).

Next, we examined whether the altered expression in the mRNA profiles were translated onto the protein level, especially that of PER2 and BMAL1 (Fig. 3E–F). We found that PER2 levels were upregulated in *Cnot1^+/−^* mice compared with expression in WT mice, especially at CT15-CT3 (subjective late night/early morning; Fig. 3E–F). BMAL1 levels were elevated during the subjective midday until the onset of the subjective night (CT6-12), even though *Bmal1* mRNA levels were not altered in *Cnot1^+/−^* livers (Fig. 3E–F).

### CNOT1 regulates the stability of *Per2* through regulating poly(A) length

**Figure 4.**
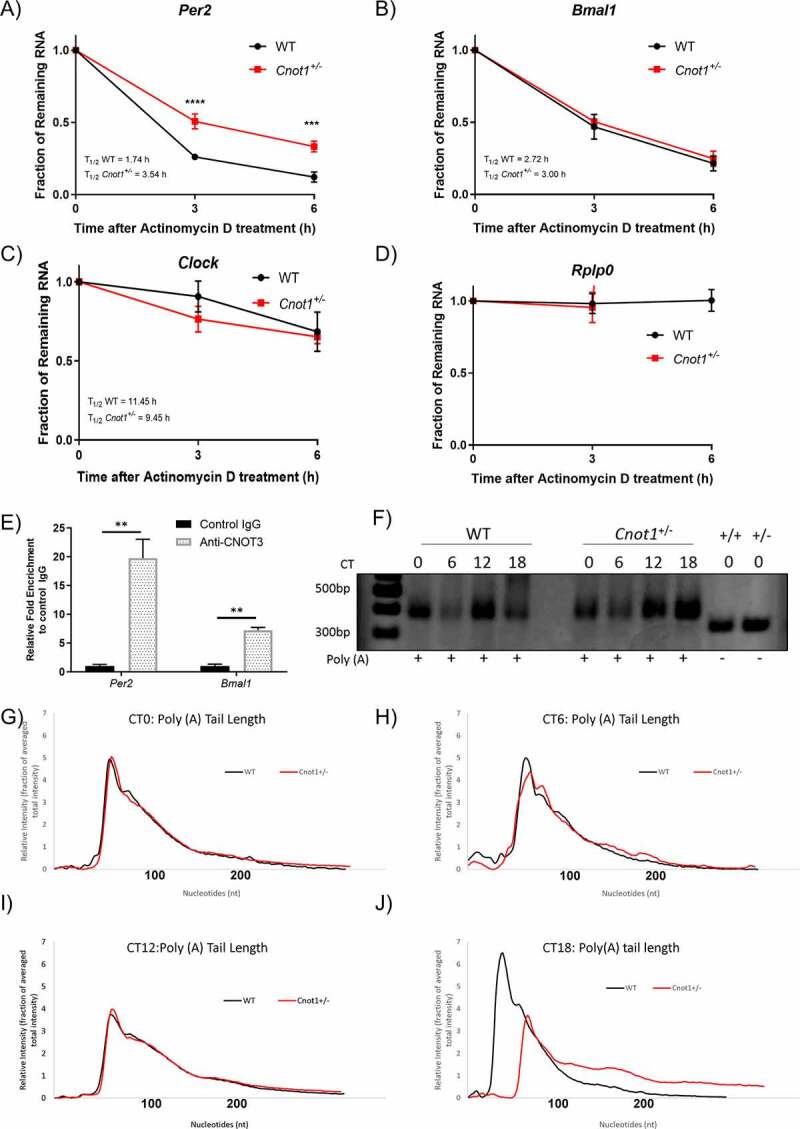
CNOT1 regulates the stability of *Per2* through regulating poly (A) length.

In the previous section, we found that *Per2* mRNA levels in *Cnot1^+/−^* mice were elevated, but only during a restricted period of the day (CT15-18), whereas protein levels were significantly elevated throughout the circadian day compared with those of WT mice. Stabilization of mRNA due to disruption of CCR4-NOT activity may lead to prolonged translation from each mRNA, and therefore could result in increased protein expression [33]. To delineate the possible mechanism for the increased mRNA expression, we examined *Per2, Bmal1, Cry1*, and *Rplp0* mRNA stability in *Cnot1*^+/-^ mouse embryonic fibroblasts (MEFs; Fig. 4A-D, Supplementary Figure 2A). Transcription was inhibited using actinomycin D (Act.D) [39] so that the decrease in mRNA over time reflects the decay rate without *de novo* transcription. In *Cnot1^+/-^* MEFs treated with Act.D, we observed that of the tested mRNAs, only *Per2* mRNA was stabilized (Fig. 4A-D). The half-life of *Per2* mRNA in *Cnot1*^+/−^ MEFs (3.54 h) was more than double that in WT MEFs (1.70 h). This suggested that *Per2* stability is regulated by the CCR4-NOT complex.

A–D). WT and *Cnot1^+/-^* MEFs were treated with 5 µg/mL of (Act.D). Relative mRNA levels of A) *Per2*, B) *Bmal1*, C) *Clock*, and D) *Rplp0* were determined by qPCR at 3 h time intervals after Act.D treatment and normalized to *Gapdh* mRNA level by ΔΔCt method. *Rplp0* was used as a positive control. mRNA level without Act.D treatment (0 h) was set to 100%. n = 3–5 for both genotypes. All values represent means ± SEM. (*p < 0.05; **p < 0.01; ***p < 0.001, unpaired t-test). E) RNA-Immunoprecipitation with anti-CNOT3 antibody and control IgG using mouse liver lysates. *Per2* and *Bmal1* mRNAs in immune complexes were analysed by qPCR (n = 3 mice for each group; *p < 0.05; unpaired t-test). Values are means ± SEM. F) Poly(A) tail length of *Per2* changes throughout the day. Representative gel of the Poly(A) tail length of *Per2* mRNA in liver of WT and *Cnot1^+/−^* mice collected every 6 h under constant darkness measured by poly(A) tail length assay (PAT). The last two lanes indicate gene specific fragment. G-H) Distribution of poly(A) tail length measured using an Agilent Bioanalyzer for G) CT0 WT vs CT0 *Cnot1^+/−^*, H) CT6 WT vs CT6 *Cnot1^+/−^*, I) CT12 WT vs CT12 *Cnot1^+/−^* and J) CT18 WT vs CT18 *Cnot1^+/−^*.

To determine whether the CCR4-NOT complex directly interacts with *Per2* and *Bmal1* mRNA in the liver, we used an anti-CNOT3 antibody to pull down the entire CCR4-NOT complex together with interacting RNAs, then performed qPCR. In anti-CNOT3 immunoprecipitates, CNOT1, CNOT3, CNOT6L, CNOT7, and CNOT8 subunits of the CCR4-NOT complex were detected, indicating that the whole complex is present (Supplementary Figure 2B). Using this approach, we detected a 19-fold and 7-fold enrichment of *Per2* and *Bmal1* mRNA, respectively, in the anti-CNOT3 mRNP immunoprecipitate relative to control IgG (Fig. 4E). This indicated that *Per2* and *Bmal1* mRNAs are likely to be direct targets of the CCR4-NOT complex.

To confirm that the stabilization of *Per2* in *Cnot1*-deficient cells was owing to a disrupted deadenylation machinery, we analysed *Per2* mRNA poly(A) tail lengths. The length of the *Per2* poly(A) tail was measured at 4 different time points in WT and *Cnot1^+/−^* liver: CT0 and CT6, during the subjective day, and CT12 and CT18, during the subjective night (Fig. 4F). Clearly, the length of the poly(A) tail of *Per2* mRNA fluctuated throughout the circadian day in both WT and *Cnot1^+/−^* (Fig. 4F). In WT livers, we observed a longer poly(A) tail in CT12 *Per2* mRNA than at other time points and shorter at CT18. In *Cnot1^+/−^*, a longer poly(A) tail was observed at CT18. A more detailed analysis and comparison of the poly(A) tail length distribution at each time point was conducted by analysing the same samples with an Agilent High-Sensitivity DNA chip (Fig. 4G–J). At CT18 and to some extent at CT6, *Cnot1^+/−^* livers contained a greater proportion of long (100 < PA) poly(A) tailed *Per2* mRNA than WT livers (Fig. 4H and 4J). At CT12 and CT0, they exhibited no change in the *Per2* mRNA poly (A) tail population (Fig. 4G-I). *Per2* poly(A) tail size distribution (Supplementary Table 1) shows that most mRNAs (53.9–87.1%) in WT liver have a poly(A) tail of less than 100 nt (mean length = 59.5 nt), while 10–32% of transcripts have a poly(A) tail between 100 nt and 200 nt (mean length = 137 nt), and only a very small fraction (2–13%) have a poly(A) tail of longer than 200 nt (mean length = 239 nt). At CT18 in WT liver, we see the highest percentage (87%) of *Per2* mRNA with a poly(A) tail shorter than 100 nt (mean length = 44 nt) compared to other time points, and only 12.9% of the transcripts have long poly(A) tails (≥100 nt). In WT, it appears that at CT18 contains the shortest tail population compared to all time points examined. Similarly, in the *Cnot1^+/-^* livers, we did not observe huge differences in poly(A) tail length except at CT12 and CT18. In contrast to WT at CT18, we observed that only 46.9% of the transcripts had a poly(A) tail shorter than 100 nt (mean length = 65 nt), while the remaining majority (53.1%) have a poly(A) tail longer than 100 nt (mean length = 142 nt). CNOT1 deficiency at CT18 specifically increased the size of short tailed poly(A) tail *Per2* species from 44 nt to 65 nt on average but did not affect the length of long poly(A) tailed transcripts (≥100 nt). This change in poly(A) tail length dynamics suggests that the increase in mRNA stability observed for *Per2* mRNA in *Cnot1*-deficient cells could be due to an elongated poly(A) mediated by disrupted deadenylase activity.

### BRF1 (ZFP36L1) binds to the AU-rich area of *Per2* 3’UTR and regulates its stability

**Figure 5.**
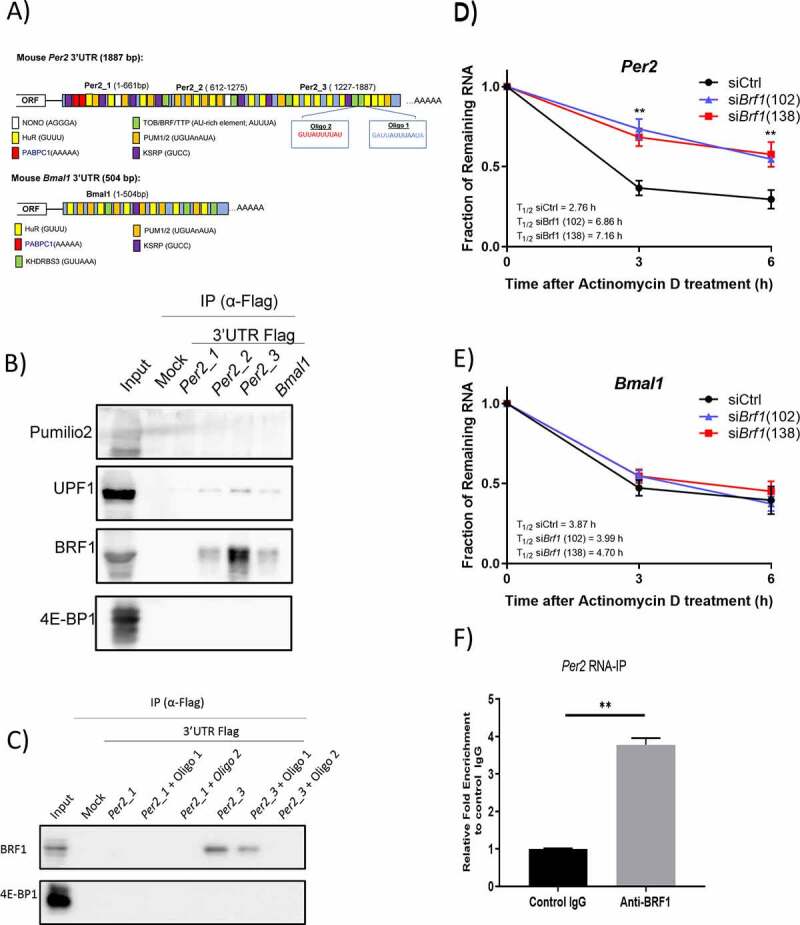
BRF1 binds to Per2 and regulates its stability.

The function of the CCR4-NOT deadenylase complex relies on its recruitment to the 3’untranslated region (UTR) of its mRNA targets through direct interaction with RNA-binding proteins (RBPs). It is well-established that the 3’UTR frequently contains multiple *cis*-acting elements that can influence mRNA stability and translation efficiency [40]. Therefore, we aimed to identify which of the known CCR4-NOT-interacting proteins bind to the 3’UTR of clock genes *in vivo*, primarily *Per2* and *Bmal1* mRNA. Analysis of the 3’UTR of *Per2* mRNA revealed that it contains multiple *cis* acting elements, including the well-studied AU-rich element (AREs) AUUUA, while *Bmal1* 3’UTR lacked AREs (Fig. 5A). To identify RBPs interacting with 3’UTR of *Per2* and *Bmal1* mRNA, we synthesized the 3’UTR *in vitro* and conjugated a Flag peptide to their 3’-ends, as previously described [41] and performed immunoprecipitation with Flag-tagged 3’UTR RNA bait. The *Per2* mRNA 3’UTR was divided into three constructs ‒ *Per2*_1, *Per2*_2, and *Per2*_3 ‒ because the technique used for fusing a Flag tag has a size limitation. The three different constructs each contained AU-rich elements (Fig. 5A). We prepared total protein lysates from WT mice liver and incubated them with Flag-tagged *Per2* and *Bmal1* mRNA 3’UTR for 1 h at 4°C. RNA-RBP complexes were purified by anti-FLAG M2 Affinity beads and analysed by immunoblotting (Fig. 5B). Of the examined CCR4-NOT-interacting RBPs, we identified BRF1(ZFP36 L1), a member of the TTP family of ARE-binding proteins, in Flag-tagged *Per2* 3’UTR immunoprecipitates. We found that BRF1 predominantly bound to the *Per2*_3 construct over *Per2*_2 and was not detected in *Per2*_1 immunoprecipitates. This indicated that the BRF1 binding site was within the *Per2*_3 region. BRF1 was also immunoprecipitated with the *Bmal1* 3’UTR, but only modestly (Fig. 5B). Similarly, UPF1, which is involved in nonsense-mediated decay (NMD), was bound, but to a lesser degree, to both *Bmal1* 3’UTR and *Per2* 3’UTR (Fig. 5B).

A) Schematic of the 3’UTR of *Per2* and *Bmal1* mRNA with predicted *cis* elements. B) Mouse liver lysates were incubated with Flag-tagged*Per2 mRNA 3’UTR (Per2_1, Per2_2, Per2_3) and Bmal1 3’UTR* bait RNAs, immunoprecipitated with FLAG M2 affinity beads, and analysed by immunoblotting with antibodies against Pumilo2, UPF1, BRF1, and 4E-BP1(negative control). Lane marked as ‘-ve’ is liver lysate without RNA bait but incubated with FLAG M2 affinity beads. C) Identification of BRF1-interacting sequences in *Per2* mRNA 3’UTR. Mouse liver lysates were incubated with Flag-tagged*Per2* mRNA 3′UTR bait RNA in the presence or absence of 12 bp of complementary LNA-oligonucleotides for 1 h. Immune complexes were analysed by immunoblotting with antibodies against BRF1 and 4E-BP1. D) RNA-Immunoprecipitation with anti-BRF1 antibody and control IgG using mouse liver lysates. *Per2* mRNA enrichment in immune complexes were analysed by qPCR (n = 3 mice for each group *p < 0.05; unpaired t-test). Mean ± SEM. E–F) HEK293 cells were transfected with control, *Brf1* (102) and *Brf1* (138) siRNA for 48 h, and then treated with Act.D. Relative mRNA levels of E) *Per2* and B) *Bmal1* were determined by qPCR at 3 h time intervals after Act.D treatment and normalized to *Gapdh* mRNA level by ΔΔCt method. mRNA levels without Act.D treatment (0 h) was set to 100%. n = 4 for both conditions. All values represent means ± SEM. (*p < 0.05; **p < 0.01; ***p < 0.001, unpaired t-test).

To identify which of the sequences in the *Per2*_3 construct is bound by BRF1, we performed a sequence-specific competition experiment using 12 base pairs (bp) locked nucleic acid (LNA) oligonucleotides (Fig. 5A,C). We designed LNA oligos targeting two AREs: Oligo1 ‒ GAUUAUUUAAUA and Oligo2 ‒ GUUAUUUUAU (Fig. 5A). Mouse liver lysates were incubated with Flag-tagged *Per2*_3 3’UTR and Flag-tagged *Per2*_1 3’UTR (used as a negative control) constructs in the presence or absence of the 12 bp complementary LNA-oligonucleotides in the same manner as shown in Fig. 5B and analysed by immunoblotting against BRF1 (Fig. 5C). Binding of BRF1 to the *Per2*_3 construct was inhibited by competition from Oligo2 and not Oligo1, indicating that binding preferentially occurred at the GUUAUUUUAU sequence. Binding of BRF1 was also reduced by competition from Oligo1 but not to a similar degree as Oligo2.

BRF1 has been reported to interact directly with CNOT1 and recruit the CCR4-NOT complex to its target mRNA and destabilizing it [41,42]. To examine the effect of BRF1 on *Per2* mRNA stability, we treated HEK293 cells with Act. D after knockdown of *Brf1* using siRNA (Fig. 5D-E). HEK293 cells were used due to ease of transfection in comparison to MEFs. A knockdown of >80% of endogenous BRF1 was achieved by targeted RNAi (Supplementary Figure 3A). As shown in Fig. 5D, *Per2* mRNA levels were stabilized in *Brf1* siRNA-treated cells (T_1/2_ = 7.01 ± 0.15 h) compared with Control siRNA-treated cells (T_1/2_ = 2.76 ± 0.52 h) with a 2.5-fold increase in half-life.

The destabilization function of BRF1 is specific to *Per2* mRNA as *Bmal1* mRNA stability and half-life remained unchanged in *Brf1* siRNA-treated cells HEK293 (Fig. 5E). Together, these results suggested that BRF1 regulates the stability of the *Per2* mRNA through the recruitment of the CCR4-NOT complex to the ARE within the *Per2* mRNA 3’UTR. Moreover, in RIP-qPCR experiments using anti-BRF1 (Supplementary Figure 3B), *Per2* mRNA was enriched at least threefold compared with control IgG (Fig. 5F). *Bmal1* mRNA was not detected in the anti-BRF1 immunoprecipitate, corroborating the notion that BRF1 binds specifically to *Per2* mRNA *in vivo*.

### BRF1 oscillates during the circadian day

**Figure 6.**
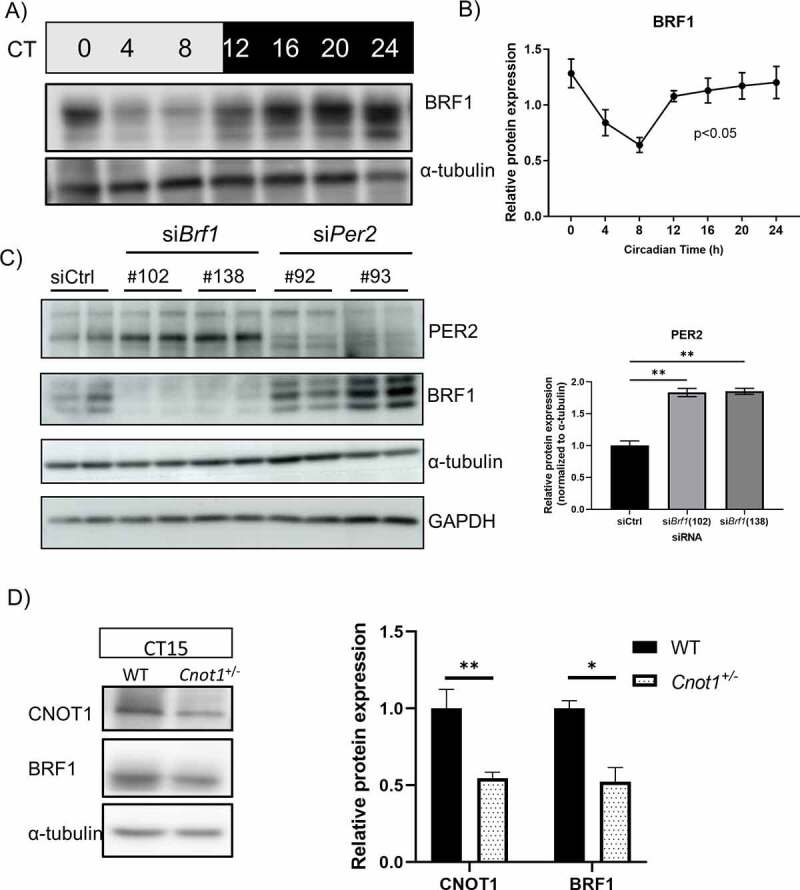
BRF1 oscillates in a circadian manner in mouse liver under DD conditions.

BRF1 binds to the 3’UTR of *Per2* mRNA, destabilizes it and promotes its decay. To elucidate whether this is relevant during the circadian cycle, we examined the BRF1 protein expression pattern. The *Brf1* gene is under circadian control and rhythmically expressed in peripheral tissue such as the heart and liver [16,43]. However, rhythmic cycling of mRNA does not necessary translate to circadian protein expression. Therefore, the BRF1 protein expression pattern during the circadian cycle is necessary to attribute a possible function for BRF1 in circadian regulation [21]. In WT mouse liver, BRF1 is expressed in a rhythmic manner (p < 0.05) with peak expression during the subjective night (CT16-24) and nadir expression during the subjective day (CT4-8; Fig. 6A, B). This circadian expression pattern of BRF1 is reciprocal to the oscillation pattern of *Per2* (Fig. 3A). Furthermore, in *Brf1* siRNA-treated HEK293 cells, we found that PER2 expression was upregulated twofold (Fig. 6C). Seeing that *Per2* mRNA was upregulated during the subjective night (CT15-18) in *Cnot1^+/-^* mice, and that BRF1 expression peaks during that time, we wanted to know if BRF1 levels are affected in *Cnot1^+/-^* mice and thereby affecting *Per2* levels. Interestingly, we found that BRF1 levels are reduced by 50% at CT15 in *Cnot1^+/-^* mice (Fig. 6D). Taken together, our data suggest that during the subjective night, BRF1 binds to *Per2* mRNA to promotes its decay via the CCR4-NOT complex to maintain its expression level. In *Cnot1^+/-^* mice, BRF1 binding to *Per2* is decreased and therefore *Per2* mRNA is stabilized.

A) Representative immunoblot against BRF1 and α-tubulin (loading control) in protein liver lysates collected every 4 h under DD conditions. B) Relative protein expression of BRF1 protein levels normalized against α-tubulin (n = 3 per time point). Values are means ± SEM. Rhythmicity was assessed by one-way ANOVA. C) Immunoblotting against BRF1, PER2, GAPDH, and α-tubulin (loading control) of BRF1 and PER2 siRNA-treated HEK293 cells and bar graph is a quantification of the blot. (n = 2 per siRNA). Values are means ± SEM (*p < 0.05; unpaired t-test). D) Representative immunoblot against CNOT1, BRF1, a-tubulin in CT15 WT and *Cnot1^+/−^* liver lysates and bar graph showing the relative protein expression (n = 3 per genotype, values mean ± SEM (*p < 0.05; **p < 0.01, unpaired t-test).

## Discussion

Post-transcriptional regulation is required for the generation of rhythmic gene expression [18,20,21,25,44–46]. Several post-transcriptional mechanisms that regulate the life cycle of (pre-) mRNA from capping, splicing, polyadenylation, stability/decay, and translation are under circadian control [47]. Because the CCR4-NOT complex is involved in all of these steps and is the major deadenylase in eukaryotes, it was important to evaluate whether a deficiency in one of the subunits would affect overall circadian behaviour [33,48]. Our results showed that in the SCN, the central pacemaker, *Cnot1* is highly expressed, and heterozygous CNOT1 mice displayed a longer circadian period. This was consistent with circadian period elongation reported in *Neurospora* strains lacking *not1*, orthologous to *Cnot1* [49]. Similar to that observed in *Neurospora*, in a genome-wide RNAi screen of human U2OS cells, *CNOT1* knockdown resulted in an elongated circadian period [50]. Interestingly, in *Cnot1*-deficient mice, CNOT9 was downregulated (Supplementary Figure 2). It has been reported that CNOT9 and CNOT1 interact with the miRISC complex to degrade miRNA targets [51,52]. Therefore, we expected to observe a similar period elongation in mice lacking *Dicer*, an enzyme responsible for generation of miRNAs. But contrary to our assumption, global *Dicer* KO mice dramatically shortened the circadian period [53]. However, using *Dicer* KO liver explants, a modest lengthening of circadian period was observed [54]. This is likely owing to tissue-specific differences in the activity of miRNAs [55]. It has been shown that one deadenylase, Nocturnin (NOC), is under circadian control but *Noc* KO mice displayed normal circadian rhythms and behaviour [56,57]. This corroborates the notion that the CCR4-NOT deadenylase complex regulates circadian rhythm and behaviour.

The control of the period length is determined by the intricate interaction of the core clock genes in an autoregulatory, transcription–translation feedback loop (TTL) [7]. Therefore, disturbances in these loops would result in lengthening or shortening of the period depending on which clock gene is altered. Several studies have reported that rhythmic PER2 levels are the key determinants in the regulation of the circadian period length [58,59]. In our study, we found that *Per2* mRNA levels were significantly affected in *Cnot1^+/−^* mice, with increased expression mainly during the subjective night. The observed expression also exhibited a phase delay in peak expression of *Per2* mRNA of approximately 3 h. However, on the protein level, we observed a significant increase in PER2 level throughout the daily cycle compared with WT mice, which was still rhythmic in nature. We believe that this increase in PER2 protein was mainly owing to the phase delay and increased mRNA expression of *Per2*. Predictive models examining mRNA oscillations suggest that mRNA stability affects the phase timing of oscillations [24]. To that end, the rate of mRNA decay of *Per2* was highly attenuated in *Cnot1*-deficient cells. We only observed an elevation of *Per2* mRNA levels in *Cnot1*^+/-^ mice after the peak time in WT mice (CT12); at this time point *Per2* mRNA levels reached their peak and entered the declining phase. As shown by Woo et al. (2009) [60], *Per2* mRNA decay kinetics are different during the rising and declining phase of the cycle (stable and unstable, respectively). Therefore, if the mRNA is stabilized it will continue to accumulate and not undergo degradation. Our data clearly indicated that this was the case in our experiments. CNOT1 deficiency resulted in reduced mRNA decay and subsequent accumulation of *Per2* mRNA transcripts, which are later translated into PER2 proteins. Mice with increased *Per2* levels showed similar period lengthening [61,62], whereas *Per2* KO mice have shorter periods [63]. Therefore, we believe that the period lengthening observed in *Cnot1*-deficient mice is a consequence of a delayed *Per2* degradation and accumulation of transcripts.

The observed stability of *Per2* mRNA is due to deadenylation deficiency as we found that the poly(A) tail length was longer in *Cnot1^+/−^* mice than in WT mice in particular at CT18 with a significant proportion of mRNAs with a poly(A) tail longer than 100 nt and a shift in size of short poly(A)-tailed *Per2* mRNAs from 44 nt to 65 nt. Deadenylation impairment is quite evident in *Cnot1* liver-specific deficient mice, in which the bulk poly(A) tail exhibited a significant shift of poly(A) tail population from 70 nt to 150 nt [37]. We believe that the main deadenylase involved in shortening the poly(A) tail of *Per2* is CNOT8, as it is the only deadenylase subunit of the complex that was downregulated in *Cnot1*^+/−^ liver (Supplementary Figure 2A–B). CNOT8 may be decreased because it is stabilized by binding to CNOT1. Therefore, with reduced CNOT1 levels, CNOT8 would undergo degradation faster in WT. We cannot rule out the involvement of CNOT7, since CNOT7 levels are not dependent on its integration into the complex [64]. Activity of CNOT7/CNOT8 are increased by binding to CCR4-NOT complex, so a reduction in CNOT1 could still affect CNOT7 activity.

**Figure 7.**
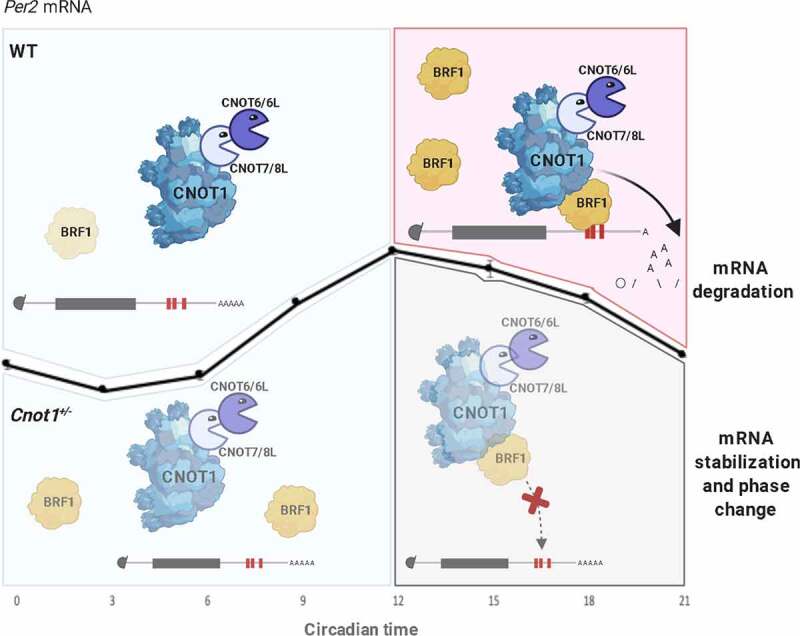
Proposed model of CCR4-NOT/BRF1 mediated decay of Per2 mRNA.

We have identified BRF1 as a *Per2* 3’UTR binding protein. BRF1 showed a reciprocal pattern of expression to that of *Per2* mRNA, with peak protein expression during the subjective night when *Per2* mRNA levels are in the declining phase. Knockdown of *BRF1* resulted in stabilizing *Per2* mRNA and upregulating its expression. We propose a model (Fig. 7) in which BRF1 binds to *Per2* mRNA during the declining phase at the subjective night and recruits the CCR4-NOT complex, shortening the *Per2* poly(A) tail length to promote its destabilization and decay, and thereby maintaining PER2 homoeostasis. In *Cnot1^+/-^* mice, BRF1 binding to *Per2* is reduced due to reduced CNOT1 and BRF1 expression during the subjective night. Decreased binding of BRF1/CCR4-NOT complex to *Per2* would result in delayed *Per2* degradation due to the slower deadenylation, and therefore a delay in *Per2* peak expression, thereby slowing the clock. Therefore, in *Cnot1*^+/−^ mice, we believe that the lengthening of the circadian period is due to an impaired deadenylation-dependent mRNA decay of *Per2*, resulting in PER2 upregulation. Previously, two other *Per2* mRNA 3’UTR binding proteins have been identified, KH-type splicing regulatory protein (KSRP) and polypyrimidine tract-binding protein (PTB) [60,62]. Both bind to the 3’UTR region of *Per2* mRNA and destabilize it. However, whether KSRP, PTB, and BRF1 interact together and work in a synergistic manner or exhibit redundant function with regard to *Per2* remains to be elucidated. BRF1 and KSRP show similar expression pattern in the liver [62] and their binding sites on *Per2* 3’UTR overlap (Fig. 5A). Therefore, we believe that they also might act cooperatively to regulate *Per2* mRNA decay by recruiting the CCR4-NOT complex. It has been shown that PTB and hnRNP Q are both required for modulating the IRES-mediated translation of Rev-erbα[65]. Therefore, it would not be surprising that an intricate interplay exists between *Per2* 3’UTR RBPs.

Suggested model showing that in WT liver, BRF1 levels are lower during the subjective morning and levels increase during the subjective night, when it binds to the 3’UTR region of *Per2*, destabilizing it by recruiting the CCR4-NOT complex, removing the tail of adenosine bases, and ultimately degrading the mRNA transcript. While in *Cnot1^+/-^* livers, BRF1 levels during the subjective night are decreased, and the BRF1/CCR4-NOT complex is not able to bind effectively to *Per2* 3’UTR thereby stabilizing *Per2* mRNA. Figure created by Biorender.com.

Interestingly, when we compared the targets of the CCR4-NOT complex (n = 4891) [37] to a list of known rhythmic genes (n = 1349) [18], only 681 genes were common (Supplementary Figure 2C). Of those genes, *Cry1, Bmal1*, and *Clock* were enriched even though their mRNA levels were not significantly altered in *Cnot1^+/-^* livers. This suggests that CNOT1 might play a different role depending on the inherent nature of the transcript. For example, *Bmal1* was found in anti-CNOT3 immunoprecipitate (Fig. 4E), but its mRNA expression and stability were unaltered (Fig. 3C, Fig. 4B) while its protein expression was upregulated (Fig. 3E-F). This suggests that the CCR4-NOT complex acts on BMAL1 either through translational repression [66] or post-translational independent of mRNA stability.

This study highlights the role of the CCR4-NOT complex in regulating circadian expression through a deadenylation-dependent mRNA decay mechanism on the *Per2* transcript. However, to fully understand the contribution of CCR4-NOT-mediated decay in circadian rhythm generation, a systematic approach is needed to examine the composition of the CCR4-NOT complex, the relevant contribution of each of the four deadenylases (CNOT6/6L/7/8), and the changing repertoire of interacting RBPs throughout the day.

## Materials and methods

### Animals

*Cnot1*^+/−^ and *Cnot1*^*fx/fx*^ mice generation has been described previously [37]. We backcrossed *Cnot1*^*+/−*^ mice with C57BL/6J mice (from which *Cnot1*^*+/−*^ were derived) for at least ten generations. Camk2a-Cre mice (B6.FVB-Tg(Camk2a-cre)2Gsc/Cnrm), which express the Cre recombinase gene undercontrol of the promoter of mouse calcium/calmodulin-dependent protein kinase II alpha (Camk2a)gene, were used to generate forebrain-specific knockout mice of *Cnot1*^67^ mice, we crossed *Cnot1* mice with Camk2a-Cre^+/-^mice. Primers used for genotyping of wild-type, knockout and floxed alleles are listed in Table 2. Mice were maintained under a 12-h light/12-h dark (LD) cycle in a temperature-controlled (22°C) barrier facility with free access to water and normal chow diet (NCD, CA1-1, CLEA Japan). All experiments were performed using 6–14 weeks old male mice. Mouse experiments were approved by the animal experiment committee at the Okinawa Institute of Science and Technology Graduate University (OIST).

### Wheel Running

Prior to experimental manipulation, animals were housed and kept under a normal 12-h light/12-h dark cycle. For wheel running experiments, mice (6–8 weeks of age) were housed individually in cages equipped with running wheels (Columbus instruments) with food and water available ad libitum. Animals were housed under a normal LD cycle until activity rhythms were stably entrained (10–14 days), and subsequently housed under DD conditions for at least 21 days. Running wheel activity was recorded using the provided software (CLAMS, Columbus Instruments). Circadian period from the running wheel activity data is calculated using the chi-square periodogram method by a freely available software developed by Dr. Roberto Refinetti’s lab [68]. Animals were housed under a normal LD cycle until activity rhythms were stably entrained (10–14 days), and subsequently housed under DD conditions for at least 21 days.

### Tissue collection

Mice were maintained under a 12-h light/12-h dark cycle for at least 2 weeks. For constant darkness experiments, mice were transferred at CT12 (7pm when lights switch off) into a constant darkness room for 36 h, sacrificing started at CT0 every 4 h for 24 h, and liver tissue was flash-frozen in liquid nitrogen and stored at −80°C until use. For transgenic mice, tissue was collected every 6 h in the same manner as above.

### Laser microdissection of the SCN

Laser microdissection of the SCN was performed as described previously [69]. Briefly, mice were killed by cervical dislocation, retinas were removed under infrared light, followed by brain excision in normal light, and frozen on dry ice. 30 µm thick coronal brain sections were prepared using a cryostat microtome and mounted on POL-membrane slides. Brain sections were fixed for 3 min in an ice-cold mixture of ethanol and acetic acid, then rinsed in ice-cold water and stained for 30 s in ice-cold water containing 0.05 vol% toluidine blue, followed by two washes in ice-cold water. Slides were quickly air dried at room temperature until the moisture decreased and then mounted on the LMD7000 device. SCN regions were microdissected and lysed in Trizol reagent (Invitrogen), and total RNA was purified using the RNeasy micro kit (Qiagen).

### In situ hybridization

*In situ* hybridization analysis was performed as described previously [70] with the following gene-specific probes: for Per1, the anti-sense probe covering nucleotides 812–1651 of the Per1 mRNA (Genbank, NM_011065) and nucleotides 4331–4909 Cnot1 mRNA (GenBank:NM_153164.4). Briefly, brains were sectioned at a thickness of 40 µm from the rostral end to the caudal end of the SCN by a cryostat. Tissue sections were transferred through 2x SSC, proteinase K (1 µg/mol, 0.1 M Tris buffer [pH 8.0]; 50 mM EDTA) for 10 mins at 37°C, 0.25% acetic anhydride in 0.1 M triethanolamine for 10 mins, and 2 × SSC for 10 mins. The sections were then incubated in the hybridization buffer [55% formamide, 10% dextran sulphate, 10 mM TrisHCl (pH 8.0), 1 mM EDTA (pH 8.0), 0.6 M NaCl, 0.2% N-laurylsarcosine, 500 μg/mL tRNA, 1 × Denhardt’s, 0.25% SDS, and 10 mM dithiothreitol (DTT)] containing radiolabeled riboprobes for 16 h at 60°C. Following a high-stringency post-hybridization wash, the sections were treated with RNase A. Air-dried sections were exposed to X-ray films (Kodak Biomax) and imaged.

### Antibodies

Antibodies against the following were used: Cnot1, Cnot3, Cnot6, Cnot6l, Cnot7, and Cnot8 (mouse monoclonal antibodies; generated by Bio Matrix Research and Research Center for Advanced Science and Technology, The University of Tokyo), BRF1/2 (#2119; Cell Signalling Technology), KSRP (A302-021A; Bethyl Laboratories), α-tubulin (#T9026; Sigma), PER2 (PER21-A, Alpha diagnostic) for mouse tissue, PER2 (STJ115134, St. John’s Laboratory) for HEK293T cells, BMAL1 (A302-616A; Bethyl Laboratories), 4E-BP1 (#9644S, Cell Signalling Technology) and GAPDH (#2118; Cell Signalling Technology).

### Quantitative PCR

Total RNA was isolated from liver using Isogen II (Nippongene), and cDNA was generated with SuperScript Reverse Transcriptase III (ThermoFisher Scientific) as described previously [31]. cDNA was mixed with primers and SYBR Green Supermix (Takara) and analysed with a Viia 7 sequence detection system (Applied Biosystems). Relative expression of mRNA was determined after normalization to the *Gapdh* level using the ∆∆Ct method. Primers are listed in Table 1.

### Western blotting

Western blotting was performed with enhanced chemiluminescence (Amersham Bioscience) as described previously [71]. Briefly, 100 mg of liver were homogenized in TNE buffer (50 mM Tris-HCl (pH 7.5), 150 mM NaCl, 1 mM EDTA, 1% NP40, and 1 mM PMSF) and centrifuged twice at 12,000RPM for 15 min at 4°C. Isolated protein lysate was quantified using a ThermoFisher BSA assay kit. Lysates in SDS sample buffer were subjected to SDS-polyacrylamide gel electrophoresis and electrotransferred onto polyvinylidene difluoride membranes. Protein bands were detected with appropriate antibodies and analysed with ImageQuant software using an Image Analyser LAS 4000 mini (GE Healthcare, Tokyo). Sequential probing of the membranes with a variety of antibodies was performed after inactivation of HRP with 0.1% NaN_3_, according to the manufacturer’s protocol.

### Poly(A) tail assay

The Poly(A) tail length of *Per2* mRNA was measured using Poly(A) Tail-Length Assay Kit (Affymetrix) according to the manufacturer’s protocol. Briefly, 1 µg of total RNA was incubated with poly(A) polymerase in the presence of guanosine (G) and inosine (I) residues to add the GI tail at the 3’-ends of poly(A)-containing RNAs. cDNA was generated with PAT (PCR poly(A) test) universal primer and reverse transcriptase using GI-tailed RNA as a template. PCR amplification was performed with gene-specific and PAT universal primers and HotStart-IT Taq DNA polymerase.

For PCR amplification of *Per2*, we used the following gene-specific primers:

Forward: 5’ – TGCTAAGAAGTTGACTTCCTAGG **–** 3’

Reverse: 5’ – TAAAAATATACTTGCTTTATTTAACAATTTTCTAAAAGGC – 3’

Poly(A) tail length was quantified with an Agilent High-Sensitivity DNA Kit (Agilent Technologies) according to manufacturer’s protocol using an Agilent 2100 Bioanalyzer (Agilent Technologies).

### Mouse embryonic fibroblasts (MEFs)

MEFs were prepared from E14-14.5 embryos extracted from pregnant WT mice that were mating with *Cnot1^+/–^* mice. The pregnant mice mothers were anaesthetized using isoflurane and euthanized by cervical dislocation, then the embryos were retrieved. The head and internal organs were carefully removed and discarded. Then, embryos were gently dissociated in 0.25% trypsin (Gibco, 15,090,046) at 37°C for 10–15 min to get a homogenous suspension. Afterwards, the cells were plated on tissue culture flask in Dulbecco’s modified Eagle’s medium (DMEM) high glucose (FujiFilm cat. no. 043–30,085) containing 10% foetal bovine serum (FBS, Gibco cat. no. 10,270–098) and 100 U/mL Penicillin-Streptomycin (Gibco cat. no. 15,140,122) cultured at 37°C in a dry incubator with 5% oxygen until confluency.

### Cell culture transient transfection:

HEK293T cells were cultured in DMEM containing 10% foetal bovine serum (FBS). For transient transfection, HEK293T cells were transfected using Lipofectamine RNAIMAX Transfection Reagent, according to the manufacturer’s protocol. For half-life measurements, HEK293 cells were transfected with human BRF1 siRNAs with the following sequences (Catalogue Number HSS101102 [BRF1 (102)], HSS186137 [BRF1 (137)], HSS186138 [BRF1 (138)]); PER2 siRNAs (Catalogue Number HSS113092 [PER2 (92)], HSS113093 [PER2 (92)]) and control siRNA for 48 h (ThermoFisher Scientific). Cells were then treated with 5 mg/mL Actinomycin D (WAK0) for a total of 6 h, and samples collected at 0, 3, 6 h after treatment.

### Preparation of bait RNA and analysis of RBPs

Mouse *Per2* 3’UTR (1887bp) and mouse *Bmal1* 3’UTR (504 bp) were cloned into the pGL3 control vector using primers listed in Table 3. The addition of the Flag-tagged and generation of Flag-tagged mouse *Per2* 3’ -UTR (1887 bp) and *Bmal1* 3’-UTR (504 bp) bait RNA were generated as described previously[41]. For identification of *Per2* 3’-UTR and *Bmal1* 3’-UTR binding proteins, livers from WT mice were solubilized in TNE buffer for 30 min at 4°C. Lysates were incubated with 10 pmol Flag-tagged 3’-UTR bait RNA for 1 h at 4°C with rotation and then incubated with ANTI-FLAG M2 Affinity gel for 1 h at 4°C with rotation. 1XSDS sample buffer was used for elution of immunoprecipitate RBPs.

For sequence-specific competition assay, all antisense-oligonucleotides against mouse *Per2* mRNA were fully LNA modified and were purchased from Gene Design: Oligo-1 (5′- GATTATTTAATA −3′), Oligo-2 (5′- GTTATTTTATGA −3′).100 pmol of oligonucleotides were incubated with 10 pmol of Flag-tagged *Per2*_2 3’UTR and *Per2*_3 3’UTR bait RNA for 1 h at 4°C followed by incubation with ANTI-FLAG M2 Affinity gel for 1 h at 4°C with rotation. The bait RNA-protein complex was lysed in SDS-sample buffer, and bait RBPs were analysed by immunoblotting. ‘

### RNA immunoprecipitation (RIP) assay

Livers collected from WT mice were homogenized in RNAase and protease inhibitor containing-TNE buffer (50 mM Tris-HCl (pH 7.5), 150 mM NaCl, 1 mM EDTA, 1% NP40, RNAase and 1 mM PMSF) and centrifuged twice at 130,000 g for 15 min at 4°C to remove debris. 100 µl of the lysates were set aside to be used as total input RNA. Protein concentrations were quantified using a Pierce BCA assay kit (ThermoFisher cat. no 23,225) and 100 µg of protein lysates were incubated with 2 mg of Cnot3 antibody (Biomatrix), 2 µg of mouse IgG (Santa Cruz), 2 µg of BRF1 (Cell Signalling) and 2 µg Rabbit IgG (Santa cru) for 1 h at 4°C with end-over-end rotation. Afterwards, 1.2 mL of Dynabeads Protein G (Invitrogen cat. no. 10007D) were added for 2 h at 4°C with end-over-end rotation. mRNAs were then immunoprecipitated and isolated using Isogen II (Nippon gene cat. no. 311–07361) according to the manufacturer’s protocol. cDNAs were reverse-transcribed from all of RNA using SuperScript Transcriptase III (ThermoFisher cat. no. 18,080,093) and Oligo (dT) _12–18_ Primer (ThermoFisher cat. no. 18,418,012) according to the following conditions (50°C for 1 h and 70°C for 15 min). qPCR reactions on diluted input and IP cDNAs were carried out with primers against endogenous mouse *Per2* and *Bmal1* using TB Green™ Premix Ex Taq™ II (Takara cat. no. RR820) with RoxII reference dye as internal control and a Viia7 machine (Applied Biosystems). The relative expression data were analysed by the ΔΔCt fold change method.

### Immunoprecipitation (IP) assay

Liver tissue was collected and then homogenized and solubilized in protease inhibitor containing-TNE buffer (50 mM Tris-HCl (pH 7.5), 150 mM NaCl, 1 mM EDTA, 1% NP40, and 1 mM PMSF), solubilized for 30 min at 4°C and then debris removal through centrifugation twice at 130,000 g for 15 min at 4°C. 100 µl of the lysates were set aside to be used as total protein input. Protein concentrations were quantified using Pierce BCA assay kit (ThermoFisher cat. no 23,225) and 10 mg of protein lysates were incubated with 2 mg of Cnot3 antibody (Biomatrix) and 2 µg of BRF1 (Cell Signalling) for 1 h at 4°C with end-over-end rotation. Additionally, IgGs, derived from the same host as the primary antibodies, were also run in parallel with IP antibodies to be used as controls for non-specific binding. Afterwards, 1.2 mL of Dynabeads Protein G (Invitrogen cat. no. 10007D) were added for 2 h at 4°C with end-over-end rotation. Immunoprecipitated proteins were then resuspended in SDS sample buffer and underwent SDS electrophoresis and western blotting, as described above. For confirmation of protein immunoprecipitation with Dynabeads, proteins were immunoblotted with appropriate antibodies against the immunoprecipitated protein. Proteins of interest were then detected with appropriate antibodies.

### Actinomycin-D chase experiment

For half-life measurements of mRNA, cells were treated with 5 mg/mL Actinomycin D (WAKO) for a total time of 6 h and samples were collected at 0, 3, and 6 h after treatment. RNA was extracted from these cells using the methods described above. For calculation of mRNA half-lives, the intercept and the slope of the linear regression line were applied according to the formula: LN (0.5/e^intercept)/slope.

### Statistical analysis

Quantifications of Western blots and quantitative RT-PCR experiments were analysed by one-way ANOVA (Fig. 1 and Fig. 6). Quantitative RT-PCR experiments comparing genotypes and circadian time were analysed by two-way ANOVA followed by Sidak’s multiple comparison test. Other comparisons were analysed by unpaired Student’s *t* tests with Prism 7 (GraphPad Software). Values represent the mean ± standard error of the mean (SEM) and are represented as error bars. Statistical significance is as indicated.

## Supplementary Material

Supplemental Material

## Data Availability

Data are available from the corresponding author upon request.
