# Supplementary material for: CNOT1 regulates circadian behaviour through Per2 mRNA decay in a deadenylation-dependent manner
Source: RNA Biol. 2022 May 5;19(1):703–18. doi: 10.1080/15476286.2022.2071026 (PMC9090297; doi:10.1080/15476286.2022.2071026)
Supplement: Supplemental Material [file KRNB_A_2071026_SM8701.zip › Revisied Supplementary Figures.pdf]

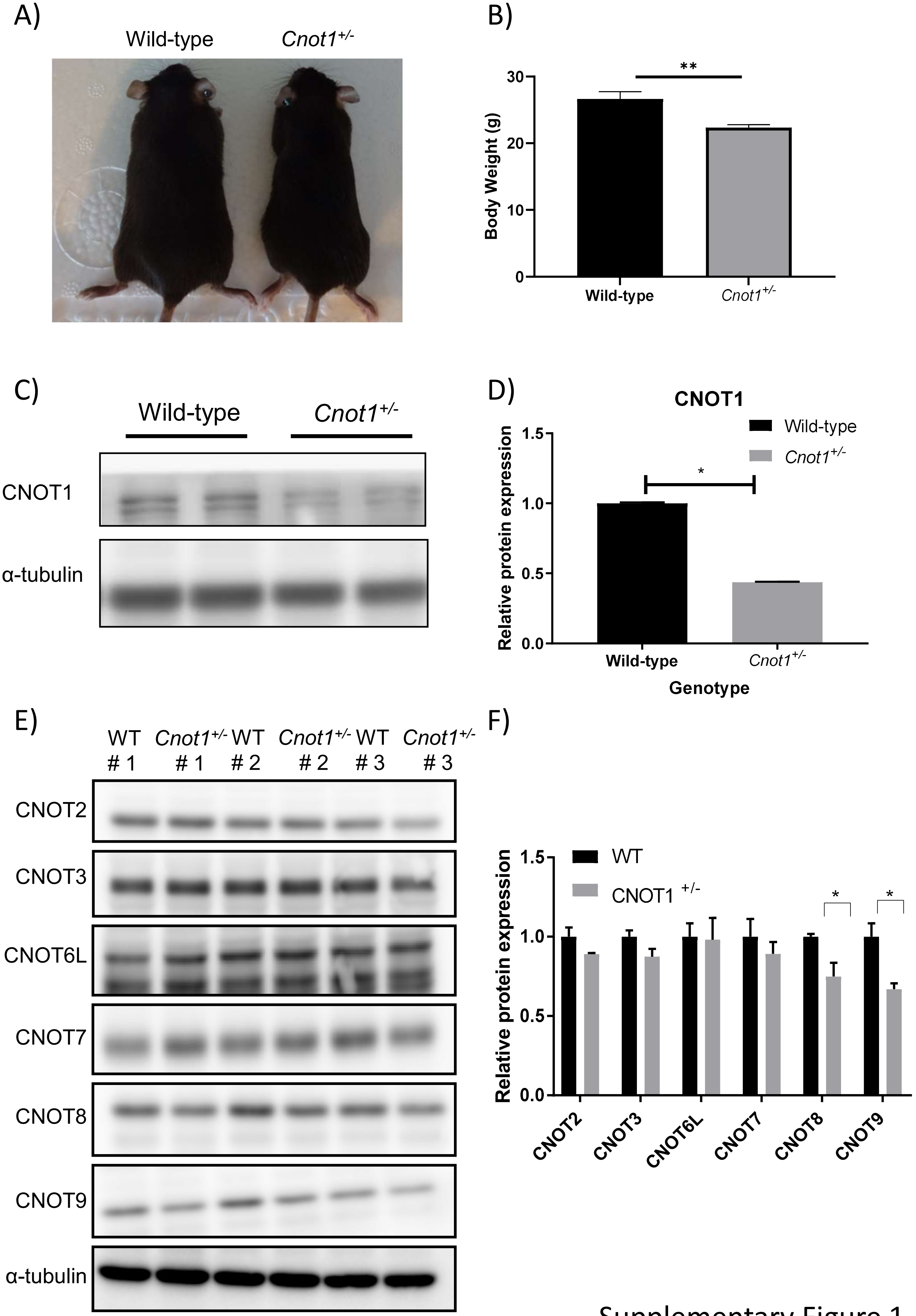

Supplementary Figure 1

A)

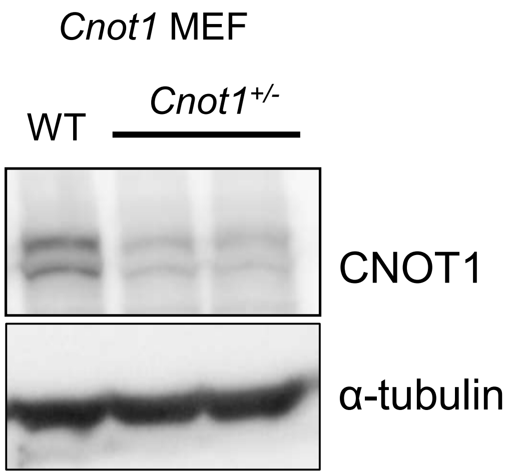

B)

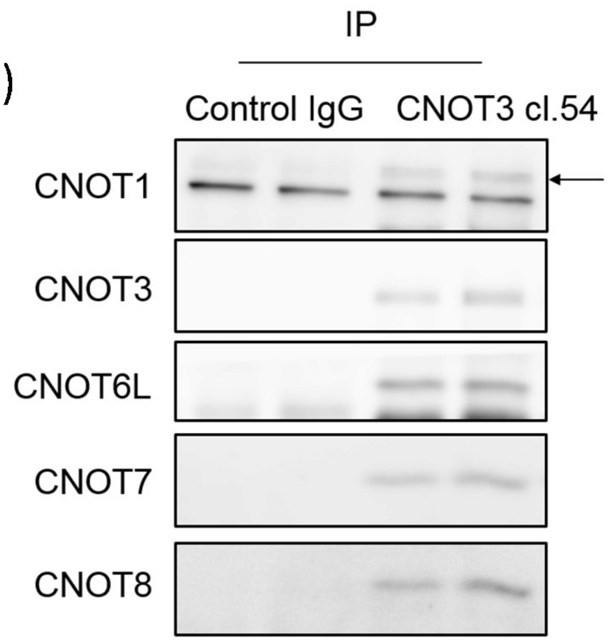

C)

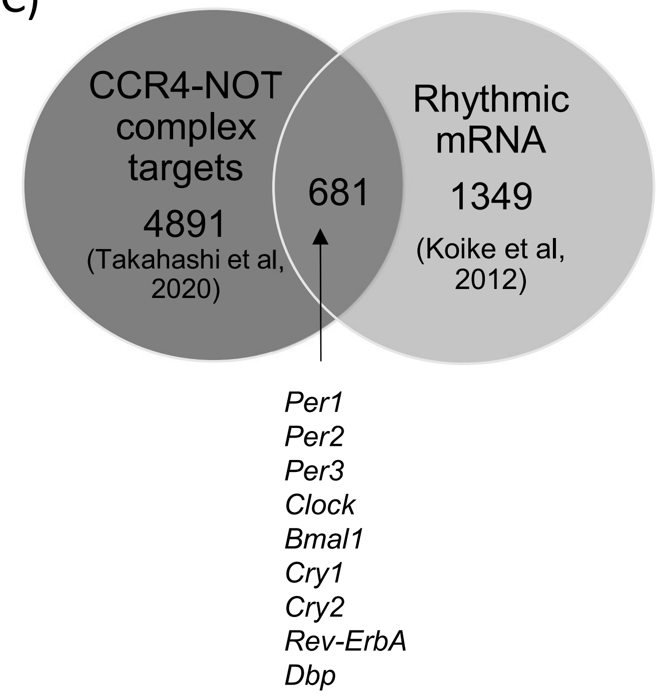

Supplementary Figure 2

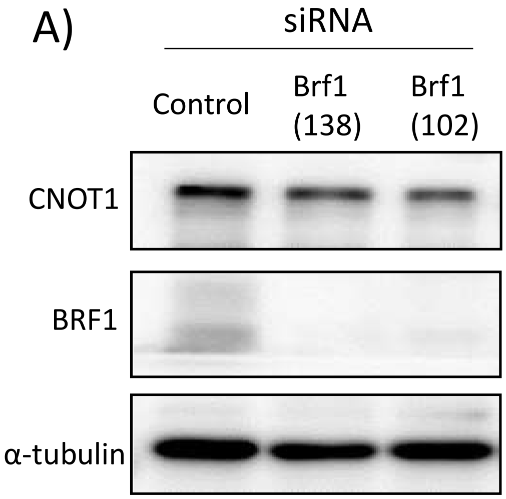

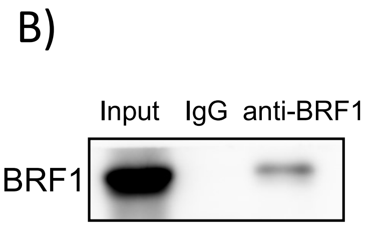

Supplementary Figure 3

	WT							
	CT0		CT6		CT12		CT18	
**Poly(A) tail length (nt)**	Percentage (%)	Average length (nt)	Percentage (%)	Average length (nt)	Percentage (%)	Average length (nt)	Percentage (%)	Average length (nt)
**PA < 100**	65.8	64	71.2	61	53.9	69	87.1	44
**100 ≤ PA < 200**	27.1	138	24.9	135	32.8	138	10.8	137
**200 ≤ PA**	7.2	239	3.8	237	13.3	239	2.1	241

	Cnot1$^{+/-}$							
	CT0		CT6		CT12		CT18	
**Poly(A) tail length (nt)**	Percentage (%)	Average length (nt)	Percentage (%)	Average length (nt)	Percentage (%)	Average length (nt)	Percentage (%)	Average length (nt)
**PA < 100**	64.9	65	68.2	62	57.5	66	46.9	65
**100 ≤ PA < 200**	25.8	138	26.4	139	28.5	139	31.2	142
**200 ≤ PA**	9.3	240	5.4	238	14	241	21.9	245

Supplementary Table 1
