# Supplementary figures and images for: CNOT1 regulates circadian behaviour through Per2 mRNA decay in a deadenylation-dependent manner
Source: RNA Biol. 2022 May 5;19(1):703–18. doi: 10.1080/15476286.2022.2071026 (PMC9090297; doi:10.1080/15476286.2022.2071026)

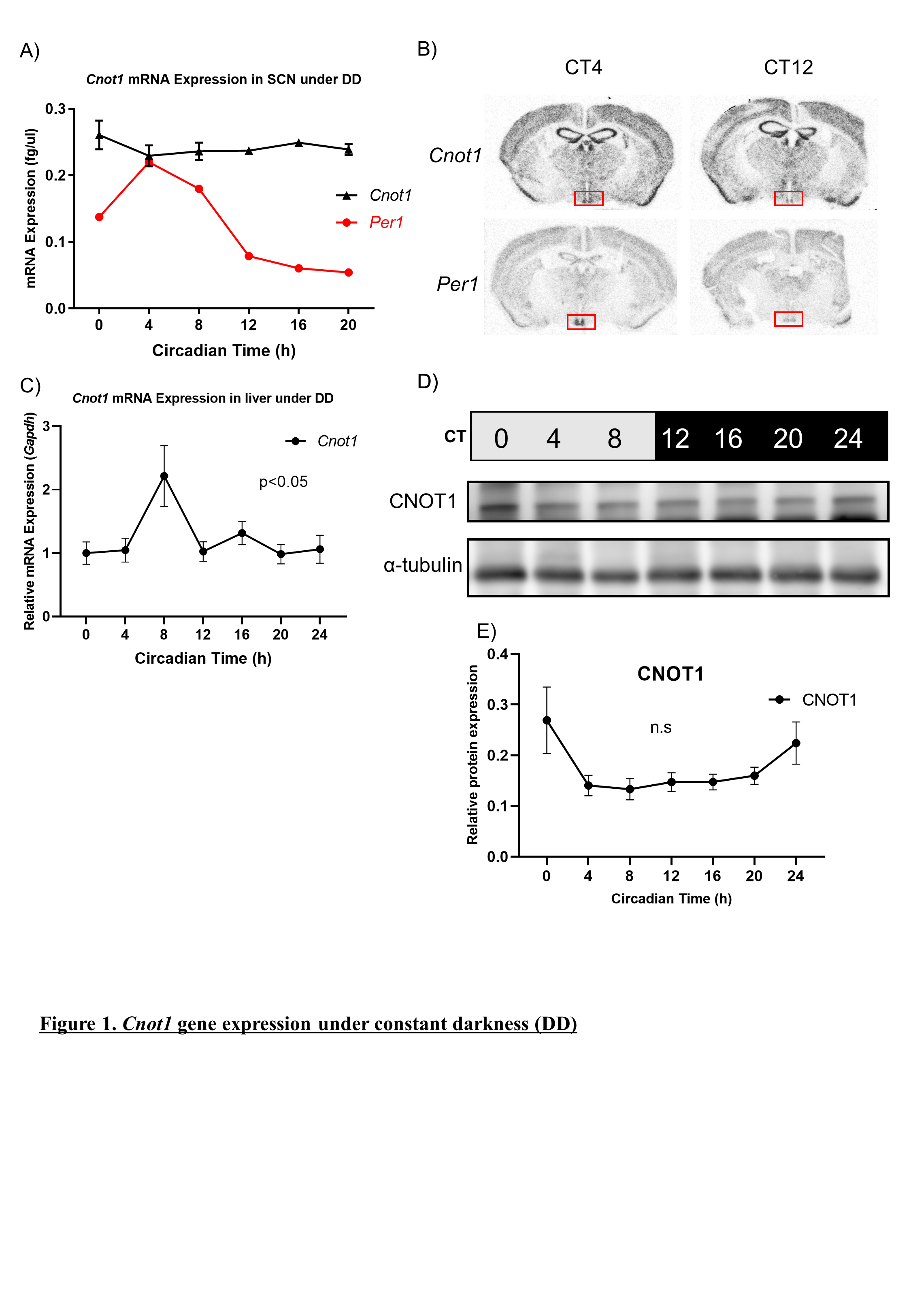

Supplement: Supplemental Material [file KRNB_A_2071026_SM8701.zip › Supplementary Figure 1 (1).TIF]

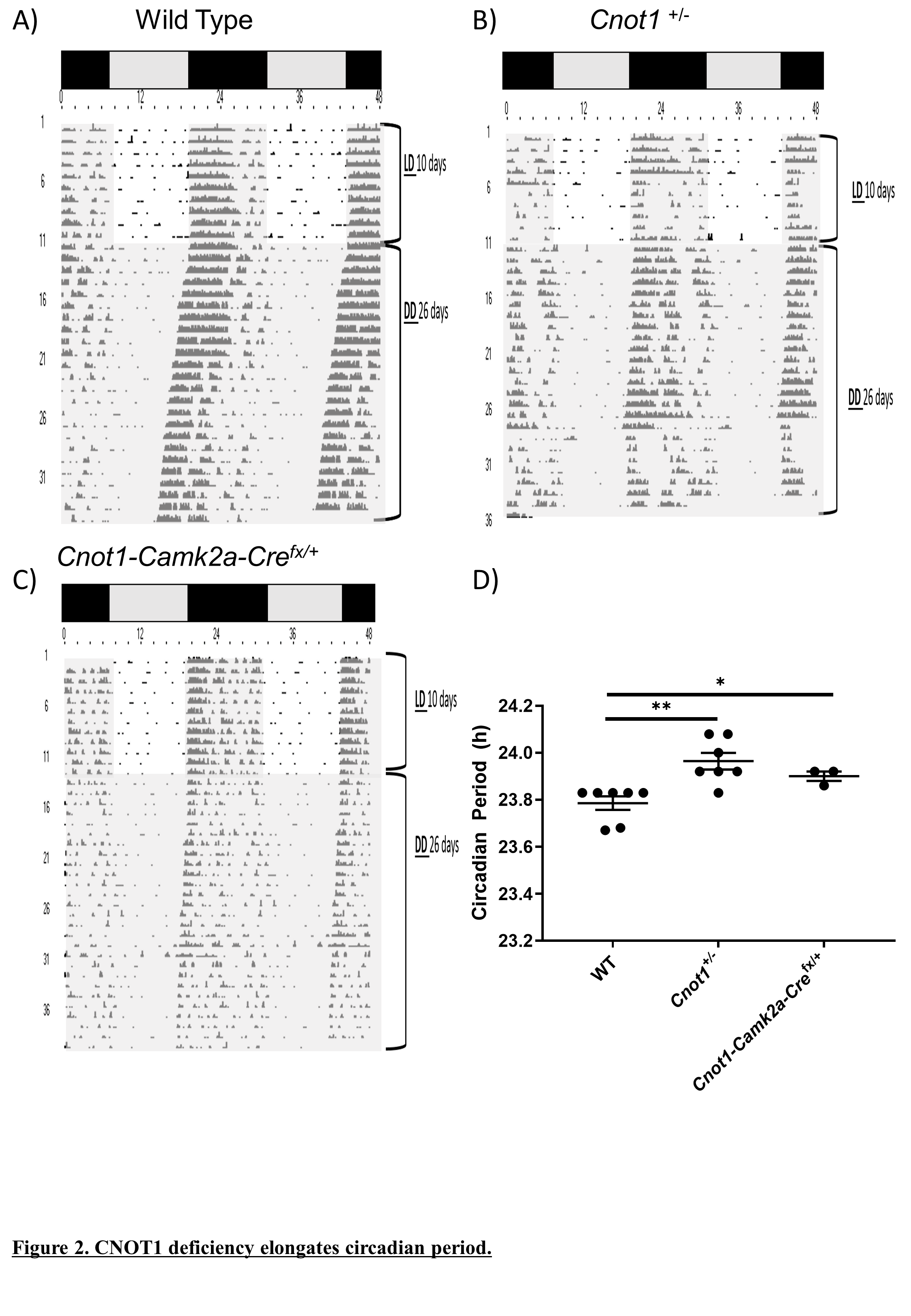

Supplement: Supplemental Material [file KRNB_A_2071026_SM8701.zip › Supplementary Figure 2.TIF]

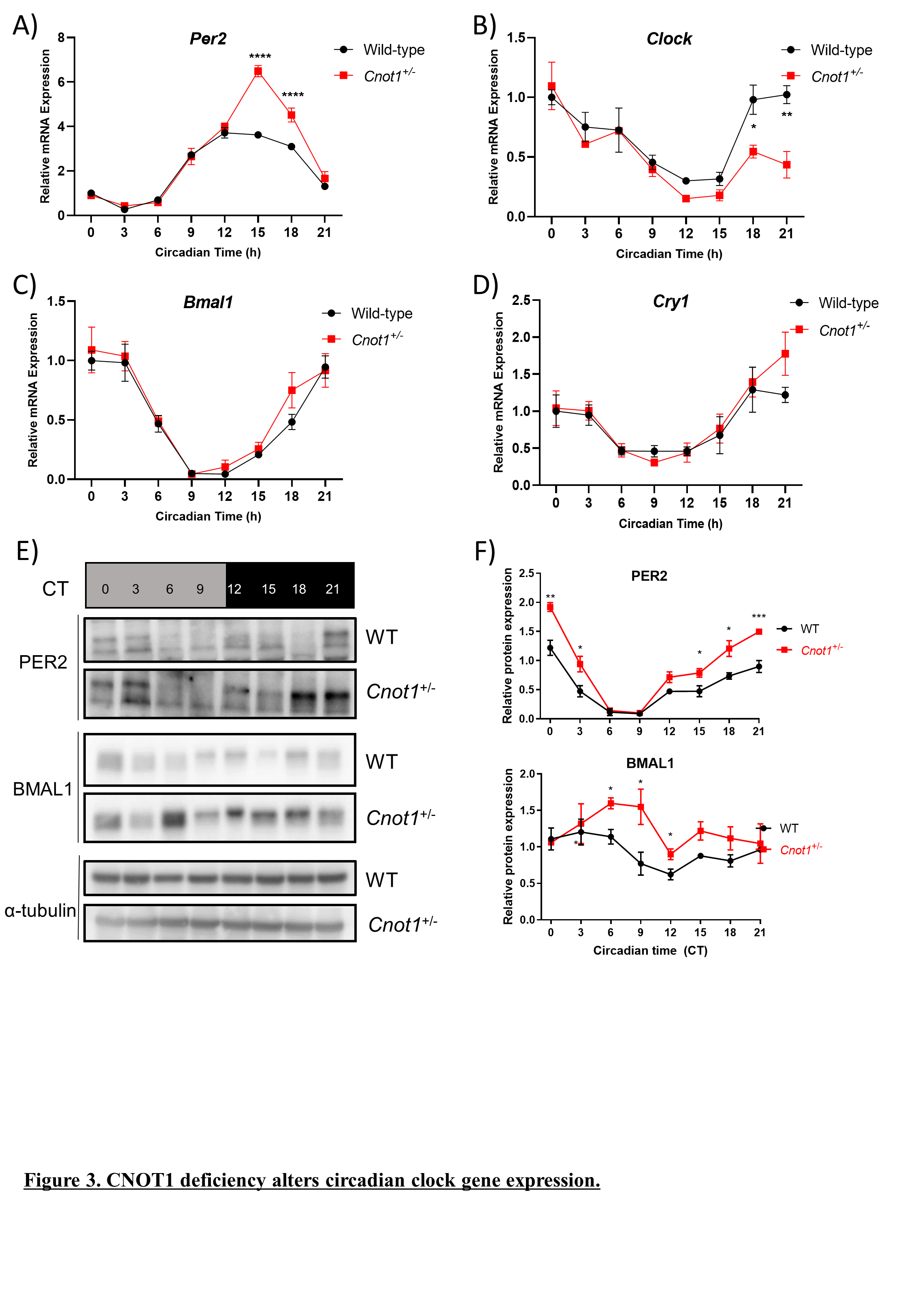

Supplement: Supplemental Material [file KRNB_A_2071026_SM8701.zip › Supplementary Figure 3.TIF]

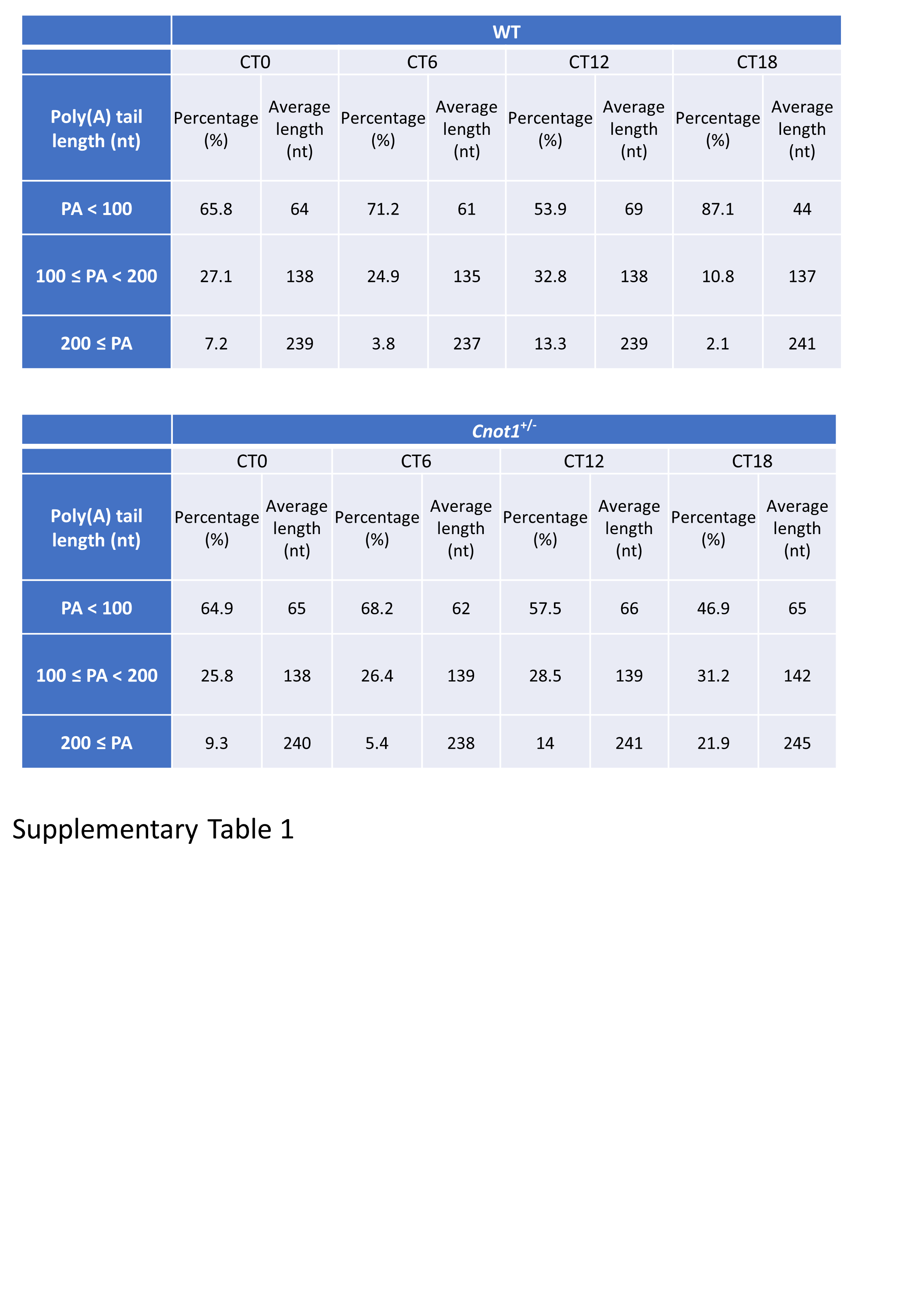

Supplement: Supplemental Material [file KRNB_A_2071026_SM8701.zip › Supplementary Table 1.TIF]
